# Lack of detection of host associated differences in Newcastle disease viruses of genotype VIId isolated from chickens and geese

**DOI:** 10.1186/1743-422X-9-197

**Published:** 2012-09-13

**Authors:** Yuyang Wang, Zhiqiang Duan, Shunlin Hu, Yan Kai, Xiaobo Wang, Qingqing Song, Lei Zhong, Qing Sun, Xiaoquan Wang, Yantao Wu, Xiufan Liu

**Affiliations:** 1 College of Veterinary Medicine, Yangzhou University, Yangzhou, Jiangsu, 225009, People’s Republic of China

**Keywords:** Genotype VIId Newcastle disease virus, Genetic, Phenotypic, Geese, Chickens

## Abstract

**Background:**

The goose is usually considered to be resistant even to strains of Newcastle disease virus (NDV) that are markedly virulent for chickens. However, ND outbreaks have been frequently reported in goose flocks in China since the late 1990s with the concurrent emergence of genotype VIId NDV in chickens. Although the NDVs isolated from both chickens and geese in the past 15 years have been predominantly VIId viruses, published data comparing goose- and chicken-originated ND viruses are scarce and controversial.

**Results:**

In this paper, we compared genotype VIId NDVs originated from geese and chickens genetically and pathologically. Ten entire genomic sequences and 329 complete coding sequences of individual genes from genotype VIId NDVs of both goose- and chicken-origin were analyzed. We then randomly selected two goose-originated and two chicken-originated VIId NDVs and compared their pathobiology in both geese and chickens *in vivo* and *in vitro* with genotype IV virus Herts/33 as a reference. The results showed that all the VIId NDVs either from geese or from chickens shared high sequence homology and characteristic amino acid substitutions and clustered together in phylogenetic trees. In addition, geese and chickens infected by goose or chicken VIId viruses manifested very similar pathological features distinct from those of birds infected with Herts/33.

**Conclusions:**

There is no genetic or phenotypic difference between genotype VIId NDVs originated from geese and chickens. Therefore, no species-preference exists for either goose or chicken viruses and more attention should be paid to the trans-species transmission of VIId NDVs between geese and chickens for the control and eradication of ND.

## Background

Newcastle disease virus (NDV) is one of the most important infectious agents in the poultry industry [1]. NDV is synonym of avian paramyxovirus 1 (APMV-1), a member of the genus *Avulavirus* in the family *Paramyxoviridae*[2]. The enveloped virus has a negative-sense, single-stranded RNA genome of 15186, 15192, or 15198 nucleotides (nts) in length, encoding six proteins: nucleocapsid protein (NP), phosphoprotein (P), matrix protein (M), fusion protein (F), hemagglutinin–neuraminidase (HN), and large protein (L) [3-5]. Phylogenetically, NDV is divided into two distinct classes (I and II). Almost all virulent NDVs belong to class II which can be subdivided into sublineages and genotypes: early sublineage of genotypes I–IV with genome length of 15186 nts, late sublineage of genotypes V–VIII with genome length of 15192 nts and genotype IX belonging to early sublineage but having a genome length of 15192 nts [6-9].

The pathogenicity of NDV isolates can be assessed by determining the mean death time (MDT) in chicken embryos, the intracerebral pathogenicity index (ICPI) in 1-day-old chicks, or the intravenous pathogenicity index (IVPI) in 6-week-old chickens [10]. MDT is used to divide virus strains into velogenic, mesogenic and lentogenic pathotypes. ICPI and IVPI are used to differentiate high pathogenic from low pathogenic NDVs. The velogenic strains are further divided into a velogenic viscerotropic pathotype, which is an acute lethal infections with necro-hemorrhagic lesions most obvious in the gastrointestinal tract, and a velogenic neurotropic pathotype, which cause predominantly respiratory and neurologic signs with high mortality [5,10,11]. The amino acid sequence at the protease cleavage site of the F protein has been postulated to be a primary molecular determinant of NDV virulence. As a rule, the virulent isolates have the cleavage site motif ^112^R/K-R-Q/K-K/R-R-F^117^, while the avirulent NDV isolates present the motif as ^112^ G/E-K/R-Q-G/E-R-L^117^[4,12-14].

A wide variety of wild, domestic and cage birds can be infected with NDV under natural and experimental conditions, while the chicken remains the most susceptible and important natural host [15]. The waterfowl are usually considered as the natural host of avirulent NDVs belonging to the class I and genotype I of class II. Moreover, the waterfowl such as geese and ducks have been thought to be resistant to strains of NDV even most virulent for chickens [11,16,17]. However, outbreaks of a novel disease entity of high morbidity and mortality with pathologic lesions of the necrosis in spleen and the extensive necrotic foci in the intestinal mucosa in goose flocks in Southern and Eastern China have been reported frequently since late 1990s [6,18-21]. The disease was described as “goose paramyxovirus (GPMV) infection” in earlier reports of Chinese literature although later work has confirmed that the concurrently emerging genotype VIId NDV in chickens is the etiologic agent of the disease [6,22,23]. Although NDVs isolated from both chickens and geese in the past 15 years are predominantly VIId viruses, published data on comparison between goose- and chicken-originated ND viruses are scarce and controversial.

Yu *et al.* first showed that the goose isolate Ch/97-1 belongs to subgenotype VIId and shares very high sequence homology with VIId NDV TW/98-4 from chicken. This suggested that Ch/97-1 might have derived or evolved from a chicken-originated VIId NDV isolate [24]. In 2003, Liu *et al.* demonstrated that there is epidemiological link between subgenotype VIId virus and the novel ND entity in geese based on the pathotypical and genotypical characterization of NDV strains isolated from geese and chickens in China during the period 1985–2001 [6]. The close phylogenicity of the goose-originated NDVs and concurrent epidemic chicken-originated NDVs has also been mentioned in other reports [18,25,26]. In contrast, Kong *et al.* demostrated the genetic difference between the chicken- and goose-originated NDVs, and used this information to establish a multiplex RT-PCR method for the differentiation between the chicken- and goose-originated genotype VIId NDVs [27]. In addition, Li *et al.* showed antigenic variation between genotype VIId NDV of goose-origin and an early lineage strain of genotype IX NDV from chicken[28]. Thus, the difference and relationship between NDVs of goose- and chicken-origin remain unclear.

To fully elucidate the genetic and pathological correlation between the goose- and chicken-originated VIId NDVs, ten entire genomic sequences and 329 complete coding sequences of individual genes from goose- and chicken-originated VIId NDVs were analyzed genetically. Two goose- and two chicken-originated VIId NDVs were randomly selected for pathological comparison in both geese and chickens with genotype IV strain Herts/33 as a reference.

## Results

### Phylogenetic analysis

**Figure 1:**
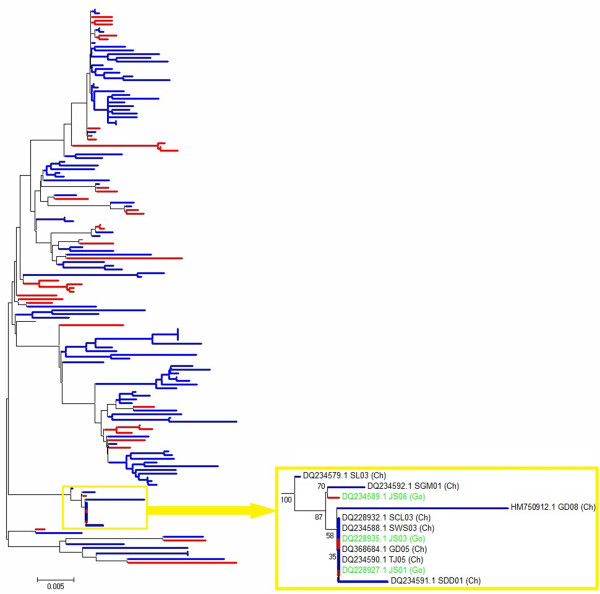
**Phylogenetic tree of 150 genotype VIId NDV strains isolated from geese and chickens based on comparison of complete HN gene sequences. ** Blue bars represent 110 chicken-originated isolates, and red bars represent 40 goose-originated isolates. Two goose-originated NDV isolates JS01 and JS03, and four chicken-originated isolates SCL03, SWS03, GD05 and TJ05 shared 100% identity. The ruler line represents the distance scale length of phylogenetic tree created by MEGA 5.0, which is 0.005.

**Figure 2:**
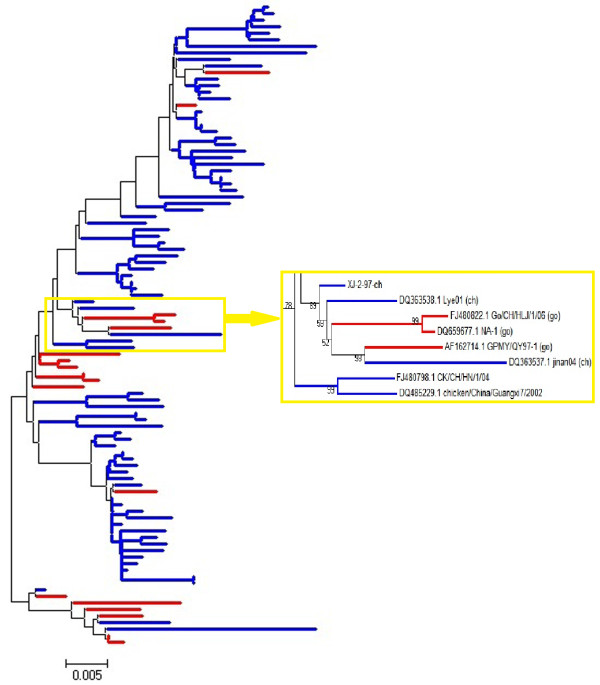
**Phylogenetic tree of 98 genotype VIId NDV strains isolated from geese and chickens based on comparison of complete F gene sequences. ** Blue bars represent 80 chicken-originated isolates. Red bars represent 18 goose-originated isolates. The ruler line represents the distance scale length of phylogenetic tree created by MEGA 5.0, which is 0.005.

**Figure 3:**
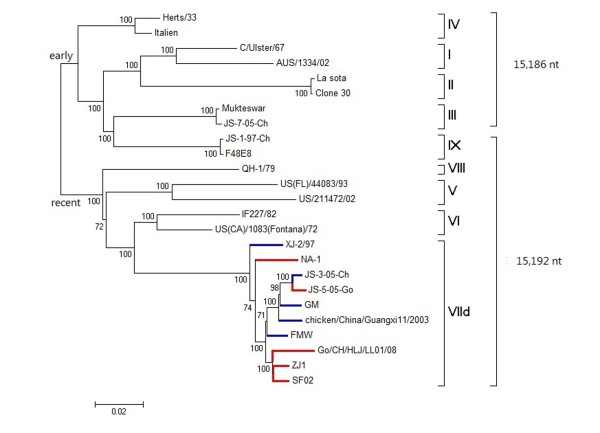
**Phylogenetic tree based on complete genome sequences of NDV strains range from genotype I to IX.** Blue bars represent 5 chicken-originated isolates, and red bars represent 5 goose-originated isolates. The ruler line represents the distance scale length of phylogenetic tree created by MEGA 5.0, which is 0.02.

Phylogenetic analysis was first performed based on the 150 complete HN gene and 98 complete F gene sequences (Figures 1 and 2, respectively). No clear interface was discovered between the goose- and chicken-originated isolates, and these two groups of isolates were clustered together and distributed randomly in the phylogenetic trees, with intermingled chicken and goose viruses. The phylogenetic trees based on the sequences of the NP, P, M, and L genes (data not shown) were similar to those based on the HN and F genes. Phylogenetic tree based on the complete genome sequences for comparing five goose- and five chicken-originated genotype VIId viruses was constructed with viruses of other genotypes as references. Five goose- and five chicken-originated VIId NDVs clustered together in phylogenetic tree on the basis of their entire genomic sequences (Figure 3).

### Sequence homology analysis

Analysis of entire genomic sequences from 18 NDVs showed that the nucleotide identity between the goose- and chicken-originated VIId NDV strains was in the range of 96.1–99.1%, while the identities within the chicken- and goose-originated groups ranged from 96.7% to 98.3% and 96% to 98.7%, respectively (Table 1). Comparatively, the nucleotide homology of genotype VIId NDVs clustered with other genotypes was 82.8–90.4%. Detailed sequence analysis of the complete genomic sequences suggested that goose- and chicken-originated strains of genotype VIId also shared very high homology in intergenic, leader and trailer sequences (Table 1). Similar results were obtained for all six protein-coding regions (Table 1). High homologies were shared in the NP, P, M, F, HN, and L genes among the genotype VIId NDVs from both geese and chickens. Comparison of 150 complete HN gene sequences from goose- and chicken-originated NDV isolates showed that six of them even shared 100% sequence identity (Figure 1).

**Table 1:** Homology comparisons of 18 complete genomic sequences of NDV strains

	**Nucleotide identity (%)**			**Amino acid homology (%)**					
	**Leader**	**Trailer**	**Total genome**	**NP**	**P**	**M**	**F**	**HN**	**L**
Between genotype VIId and other genotypes	85.5–98.5	67.3–80.5	82.8–90.4	92.9–96.5	82.6–87.9	88.8–96.2	89.4–95.5	89.7–94.9	93.9–96.3
Between Goose-originated strains of genotype VIId	96.4–100.0	93.8–100.0	96.0–98.7	98.2–99.2	96.2–98.2	96.4–99.5	97.1–98.6	96.9–98.1	97.9–99.3
Between chicken-originated strains of genotype VIId	92.7–98.2	96.5–100.0	96.7–98.3	98.8–99.4	92.7–97.5	96.2–98.6	97.7–99.1	97.2–99.3	98.5–99.1
Between goose-originated and chicken-originated strains of genotype VIId	94.5–100.0	92.9–100.0	96.1–99.1	98.4–99.6	93.9–99.2	95.9–98.9	96.9–99.1	96.7–99.3	98.0–99.1

### Comparison of amino acid sequences of individual genes

According to the nucleotide sequences, all goose- and chicken-originated NDVs belonging to genotype VIId with fusion protein cleavage site motif ^112^R/K-R-Q/K-K/R-R-F^117^. This motif is the characteristic molecular determinant of virulent NDV strains. Characteristic amino acid substitutions of VIId NDVs distinct from NDVs of other genotypes were identified based on the data from 329 complete coding sequences of individual genes from genotype VIId NDVs and 18 entire genomic sequences of NDV strains from genotypes I through IX. The results showed that all the VIId NDVs from geese and chickens shared the same characteristic amino acid substitutions (Table 2) and no amino acid residue was found to be specific for either chicken- or goose-originated viruses.

**Table 2:** **Unique residue substitutions of deduced amino acid sequences of individual genes from goose- and chicken-originated VIId NDVs**^**b**^

**Genotype**	**NP**				**P**			**F**								**HN**		**L**				
	**170**	**426**	**450**	**479**	**73**	**179**	**216**	**52**	**71**	**176**	**255**	**272**	**314**	**402**	**489**	**54**	**145**	**424**	**477**	**1545**	**1547**	**2139**
I	A	A	L	P	D	A	H	I	K	A	V	N	F	A	D	S	A	V	K	I	M	M
II						V													N			
III																G	T					
IV															N	G						
V					G	V										N	T					T
VI																R	T					
VIII																	V		R			
IX																G						
VIId (goose-originated)	V	T	F	A	E	E	R	V	R/-	S	I	Y	Y	T/-	E/-	H	I	I	S	V	V	A
VIId (chicken-originated)	V/-^c^	T	F	A	E	E	R	V	R/-	S	I	Y	Y	T/-	E/-	H	I	I	S	V	V	A

^b^ Based on data from 329 complete coding sequences of individual genes from both goose- and chicken-originated VIId NDVs, and 18 entire genomic sequences of NDV strains range from genotype I to IX. No characteristic amino acid residues from the M gene were found in NDVs of genotype VIId.

^c^ A few isolates or strains showed no change.

### Cytopathogenicity of different NDVs in chicken embryo fibroblast (CEF) cells

The five velogenic NDV strains (Herts/33 of genotype IV; chicken originated XJ-2/97 and JS-3-05-Ch, and goose originated ZJ1 and JS-5-05-Go of genotype VIId) induced similar cytopathic effects (CPE) characterized by rounding, vacuolation, syncytia formation, and cell death in CEFs monolayer, as determined by microscopic examination. In all groups, visible cytopathic changes in cell shape and in the nucleus were observed at 24 hours post inoculation (hpi). The peaks of HA titers in supernatant reached at 48–60 hpi, and the CEF monolayers were destroyed completely at 72–96 hpi. All five isolates induced extensive syncytia formation, and no significant difference in morphological changes was found between them. The description of the kinetics of the CPE is given in Table 3.

**Table 3:** Cytopathogenicity of different NDVs in CEF

**Strain**	**MDT**^**d**^	**ICPI**^**d**^	**Host**	**Virulence**	**Genotype**	**Time of the cytopathic effect observed**	**Time of HA titer peak**	**Time of complete CEF monolayer destruction**	**HA titer**
Herts/33	48.0	2.00	fowl	velogenic	IV	12–24 h	48 h	72 h	2^6^
JS-5-05-Go	49.0	1.86	goose	velogenic	VIId	12 h	60 h	72 h	2^6^
ZJ1	51.6	1.89	goose	velogenic	VIId	12 h	48 h	84–96 h	2^6^
XJ-2/97	57.6	1.94	fowl	velogenic	VIId	12–24 h	48 h	84–96 h	2^7^
JS-3-05-Ch	56.2	1.83	fowl	velogenic	VIId	12 h	48 h	84 h	2^6^

^d^ See references [7], [6], and [19].

### Clinical signs

Clinical observations are summarized in Table 4. All chickens infected with five velogenic NDV strains (Herts/33, ZJ1, JS-5-05-Go, XJ-2/97, JS-3-05-Ch) were slightly depressed, with decreased food and water consumption and had ruffled feathers at 2 days post inoculation (dpi). At 3 dpi, chickens in these five groups exhibited clinical signs including moderate to severe depression, lethargy, ruffled feathers, open-mouth breathing, anorexia, eyelid edema and diarrhea. First spontaneous deaths occurred on 3 dpi, and the other chickens were either found dead or euthanatized in a moribund state between 4 and 5 dpi. In all these five groups, mortality reached 100% on 5 dpi in chickens. No clinical signs of disease were observed in any of the non-infected controls.

**Table 4:** Clinical signs and gross lesions of SPF chickens and geese infected with different NDV strains

**Strains**	**Clinical signs first evident**	**No. leisions/total**	**Mortality**^**e**^	**Main clinical signs**	**Salient gross lesions**
				**SPF chickens**	
Herts/33	2 dpi	12/12	8/8	severe depression, lethargy, ruffled feathers, eyelid edema, diarrhea, open-mouth breathing	Spleen, Atr; Intestene, Hem; Proventriculus, Hem; Pancreas, Nec; Thymus, Hem; Larynx and trachea, Hem;
ZJ1	2 dpi	12/12	8/8	severe depression, lethargy, ruffled feathers, eyelid edema, diarrhea, open-mouth breathing	Spleen, Nec; Intestene, Hem + Nec; Proventriculus, Hem; Pancreas, Nec; Thymus, Hem; Larynx and trachea, Hem;
JS-5-05-Go	2 dpi	12/12	8/8		
XJ-2/97	2 dpi	12/12	8/8		
JS-3-05-Ch	2 dpi	12/12	8/8		
				**Geese**	
Herts/33	4 dpi	12/12	4/8	morderate depression, diarrhea, crouch, nasal discharge, eyelid edema	Spleen, Atr; Intestene, Hem, Proventriculus, Ed + Hem; Pancreas, Nec;Thymus, Hem; Larynx and trachea, Hem;
ZJ1	3 dpi	12/12	5/8	morderate to severe depression, diarrhea, crouch, nasal discharge, eyelid edema	Spleen, Nec; Intestene, Hem + Nec; Proventriculus, Ed + Hem; Pancreas, Nec; Thymus, Hem; Larynx and trachea, Hem;
JS-5-05-Go	3 dpi	12/12	6/8		
XJ-2/97	3 dpi	12/12	5/8		
JS-3-05-Ch	3 dpi	12/12	6/8		

^e^ Four birds scheduled sampled at 2 and 4 dpi in each group were reduced. Art, Atrophic; Nec, necrosis; Ed, edema; Hem, hemorrhage.

In geese infected with the four genotype VIId strains (ZJ1, JS-5-05-Go, XJ-2/97, JS-3-05-Ch), signs were first observed at 3 dpi and consisted of moderate to severe depression with anorexia, diarrhea, crouch, eyelid edema and nasal discharge. All geese exhibited obvious clinical signs from 4 dpi. Geese in these four groups died or were euthanized when moribund between 4 and 6 dpi. At 7 dpi, no goose died spontaneously or was moribund, and the clinical signs of some remaining geese began to abate. The number of geese survived up to 7 dpi in ZJ1 group, JS-5-05-Go group, XJ-2/97 group and JS-3-05-Ch group was 3, 2, 3 and 2, respectively (Table 4). In Herts/33-infected group, all geese were clinically normal at 3 dpi. From 4 dpi, eight geese exhibited clinical signs similar to what observed with VIId-infected groups. Between 5 to 6 dpi, two geese died and two geese were euthanized when moribund. By 7 dpi (end of the experiment), four goose were left (Table 4). The non-infected control geese appeared normal during the experiment.

### Gross lesions

**Figure 4:**
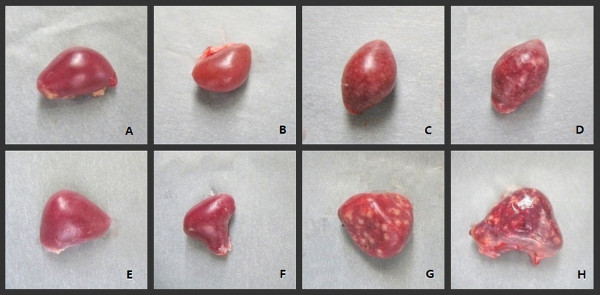
**Gross lesions on spleen of SPF chickens and geese experimentally infected with different NDV strains at 2 dpi.4A**: Spleen of chicken from control group **4B**: Atrophic spleens of chicken from Herts/33-infected group without white necrotic spots. **4C** and **4D**: Spleen of chicken infected with JS-5-05-Go and JS-3-05-Ch strains respectively, characteristic necrosis was seen in spleen. **4E**: Spleen of goose from control group. **4F**: Atrophic spleen of goose from Herts/33-infected group without white necrotic spots. **4G** and **4H**: Spleens of geese infected with JS-5-05-Go and JS-3-05-Ch strains, respectively, showing characteristic necrosis. (ZJ1 and XJ-2/97 strains induced the same gross lesion as JS-5-05-Go and JS-3-05-Ch strains, so the pictures are not shown.)

Gross findings in chickens and geese are presented in Table 4. In chickens, all the four VIId viruses (ZJ1, JS-5-05-Go, XJ-2/97, JS-3-05-Ch), irrespective of their origins, induced similar gross lesions at the same dpi. The chickens infected with the four VIId NDVs experienced the moderate to severe lesions in various organs, especially in the digestive and immune organs. Splenomegaly with severe necrotic lesions was observed throughout the spleens of almost all infected chickens (Figure 4). This change appeared as early as 2 dpi, which was prior to the development of the obvious clinical signs. Multiple foci of necrosis and hemorrhages were first presented in the small intestine of chickens infected with genotype VIId NDVs at 2 dpi, and were increased in severity up to 7 dpi with the progression of disease. In addition, the duodenum and ileum were most affected. Other lesions consisted of multifocal necrosis of the pancreas, mild congestion of the liver, hemorrhages of the thymus, larynx and trachea, hemorrhages of the proventriculus (Additional file 1) in most of these chickens between 2 and 5 dpi. Occasionally, pale kidneys with deposition of urate (Additional file 1), congestion in the brain were observed at 4 and 5 dpi. In the Herts/33-infected chickens, however, no necrotic lesion of spleens was observed during the whole experiment. The spleens were atrophic at 2 dpi (Figure 4), and became more obvious at 4 dpi. The gross lesions of the intestinal tract were also different from the VIId groups. Chickens infected with Herts/33 had gross lesions similar to those observed in VIId-infected chickens, however, main differences were observed in the spleen and intestines. In the spleen, then main difference was atrophy with lack of necrosis, whereas in the intestine multiple hemorrhages were present, but no necrotic foci were observed. Except the spleen and intestine, the gross lesions of other tissues induced by Herts/33 were similar to the four VIId strains in chickens at the same time point.

In geese, no differences were observed between groups infected with the four VIId strains. Similarly to what observed in chickens, most conspicuous lesions were present in the spleen and in the intestines (Table 4). As in chickens, Herts/33-infected geese showed lesions similar to the VIId strain-infected geese, however a significant difference was the lack of necrosis in both spleen and intestine of Herts/33-infected geese. Atrophic spleens without white necrotic spots (Figure 4), and hemorrhage in small intestine without foci of necrosis were observed in Herts/33-infected geese between 2 and 7 dpi. On other tissues, the geese infected with VIId NDVs and Herts/33 exhibited similar gross lesions at the same dpi, such as multiple necrosis foci in the pancreas, enlarged liver with congestion, thymic hemorrhages (Additional file 1), proventriculus edema with mild hemorrhages, and hemorrhage of the tracheal mucosa.

### Histopathology

**Figure 5:**
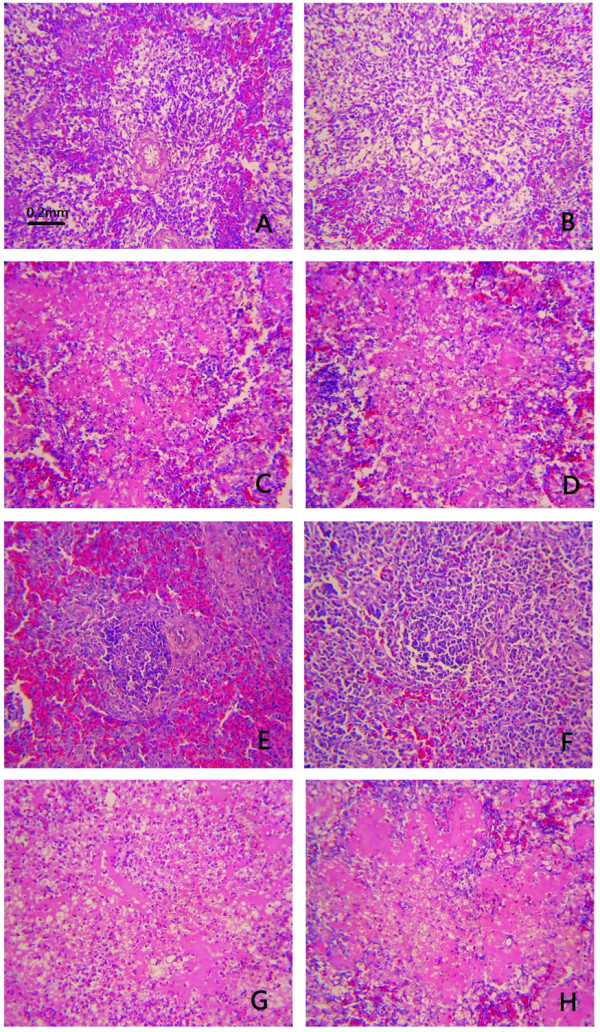
**Histopathological changes in spleens of SPF chickens and geese experimentally infected with NDVs at 4 dpi. 5A**: Spleen of chicken from control group. **5B**: Spleen of chicken infected with Herts/33, showing decreased lymphocytes in periarterial lymphatic sheath without necrosis. **5C** and **5D**: Spleens of chickens infected with JS-5-05-Go and JS-3-05-Ch strains, respectively, showing severe lymphoid necrosis. **5E**: Spleen of goose from control group. **5F**: Spleen of goose infected with Herts/33 showing no significant lesions. **5G** and **5H**: Spleen of goose infected with JS-5-05-Go and JS-3-05-Ch strains, respectively, showing severe lymphoid necrosis. (ZJ1 and XJ-2/97 strains induced the same histopathological change as JS-5-05-Go and JS-3-05-Ch strains, so the pictures are not shown.)

Results of histopathological evaluation are presented in Table 5. The microscopic findings were consistent with the gross lesions mentioned above. All the four VIId NDV strains induced very similar histological changes in multiple organs and tissues at the same dpi, and the most extensive damage was presented in the digestive and lymphoid organs in both chickens and geese. From 2 dpi, moderate to severe lymphoid depletion along with lymphoid necrosis was observed in lymphoid organs (spleen (Figure 5), bursa, and thymus) in both chickens and geese infected with VIId strains. Hemorrhage and necrosis of mucosal lymphoid tissue in the intestinal tract, necrosis in intestinal epithelium were seen starting at 2 dpi and became more severe with progression of the disease. In addition, the lesions of other tissues reached their peak at 4 dpi both in geese and chickens, such as multifocal necrosis in pancreas, necrosis of epithelial cell in proventriculus, sloughing of the epithelium in the trachea, degeneration or necrosis of hepatocytes and epithelium in the renal tubules. Acute neuronal swelling and perivascular oedema were occasionally found in chickens at 4 dpi, and in geese at 4 or 7 dpi.

**Table 5:** Distribution and intensity of histological lesions and IHC staining for NDV fusion protein

**Organs**	**Stain**	**SPF chickens**										**Geese**														
		**Herts/33**		**ZJ1**		**JS-5-05-Go**		**XJ-2/97**		**JS-3-05-Ch**		**Herts/33**			**ZJ1**			**JS-5-05-Go**			**XJ-2/97**			**JS-3-05-Ch**		
		**2 dpi**	**4 dpi**	**2 dpi**	**4 dpi**	**2 dpi**	**4 dpi**	**2 dpi**	**4 dpi**	**2 dpi**	**4 dpi**	**2 dpi**	**4 dpi**	**7 dpi**	**2 dpi**	**4 dpi**	**7 dpi**	**2 dpi**	**4 dpi**	**7 dpi**	**2 dpi**	**4 dpi**	**7 dpi**	**2 dpi**	**4 dpi**	**7 dpi**
Spleen	HE	-	+	+++	+++	+++	+++	+++	+++	+++	+++	-	-	-	+++	+++	+++	+++	+++	+++	+++	+++	+++	+++	+++	+++
	IHC	+	+++	+	+++	+	+++	+	+++	++	+++	-	++	+++	+	+++	+++	+	+++	+++	+	+++	+++	+	+++	+++
Bursa	HE	++	+++	++	+++	++	+++	++	+++	++	+++	++	+++	+++	++	+++	+++	++	+++	+++	++	+++	+++	++	+++	+++
	IHC	++	+++	++	+++	++	+++	++	+++	++	+++	+	+++	+++	++	+++	+++	++	+++	+++	++	+++	+++	+++	+++	+++
Thymus	HE	+++	+++	+++	+++	+++	+++	+++	+++	+++	+++	+++	+++	+++	+++	+++	+++	+++	+++	+++	+++	+++	+++	+++	+++	+++
	IHC	+++	+++	+++	+++	+++	+++	+++	+++	+++	+++	++	+++	+++	+++	+++	+++	+++	+++	+++	+++	+++	+++	+++	+++	+++
duodenum	HE	+	++	+	+++	++	+++	+	+++	++	+++	+	++	++	+	+++	+++	+	+++	+++	++	+++	++	++	+++	++
	IHC	++	+++	+++	+++	+++	+++	+++	+++	+++	+++	-	++	++	+++	+++	+++	+++	+++	+++	+++	+++	+++	+++	+++	+++
ileum	HE	+	++	++	+++	++	+++	++	+++	++	+++	+	++	+	++	+++	+++	+	+++	++	+	+++	+++	++	+++	+++
	IHC	++	+++	+++	+++	+++	+++	+++	+++	+++	+++	-	++	++	+++	+++	+++	+++	+++	+++	+++	+++	+++	+++	+++	+++
cecum	HE	+	++	+	++	+	+++	++	++	+	++	+	+	++	+	++	++	+	++	+++	+	+++	++	+	++	++
	IHC	++	+++	+++	+++	+++	+++	+++	+++	+++	+++	-	++	++	+++	+++	+++	+++	+++	+++	+++	+++	+++	+++	+++	+++
Pancreas	HE	++	+++	+	+++	++	+++	+	+++	++	+++	++	+++	++	+	++	++	++	+++	++	+	++	++	+	+++	+++
	IHC	+	++	+	+	+	++	+	++	+	++	+	+	++	+	+	++	+	++	++	+	+	++	+	++	++
Proventriculus	HE	+	+++	+	+++	+	+++	+	+++	+	+++	-	++	++	+	++	++	-	++	+	+	++	++	+	+	++
	IHC	+	+++	+	++	+	+++	+	++	+	++	+	+	++	+	+	++	+	++	++	+	+	++	+	++	++
Liver	HE	+	++	+	+	++	+	-	++	+	+	+	++	+	++	++	++	+	+	++	+	++	+	+	+	++
	IHC	+	+++	+	++	+	++	+	++	+	+++	+	++	+	+	+	++	+	+	++	+	++	++	+	+	++
Kidney	HE	-	+	-	+	-	+	-	+	-	-	-	-	+	+	-	+	-	-	+	-	+	-	-	+	+
	IHC	+	++	+	++	+	+	+	++	+	++	+	+	++	+	+	+	+	++	+	+	+	++	+	+	++
Lung	HE	-	+	-	-	-	-	-	-	-	+	-	+	-	-	-	+	-	-	+	-	-	-	-	+	+
	IHC	-	++	+	+	-	+	-	+	+	++	-	+	+	-	+	+	-	+	+	-	+	+	+	+	+
Trachea	HE	-	++	-	++	-	++	-	++	+	++	-	+	+	-	+	+	-	+	+	-	+	+	+	+	+
	IHC	-	+	-	+	-	+	-	+	-	+	-	+	+	-	+	+	-	+	+	-	+	+	-	+	+
Cerebrum	HE	-	+	-	+	-	-	-	-	-	+	-	+	-	-	-	-	-	-	+	-	-	-	-	+	-
	IHC	-	-	-	-	-	-	-	-	-	-	-	-	-	-	-	-	-	-	-	-	-	-	-	-	-

-, no lesions/negative for NDV fusion protein by IHC; +, mild lesions/< 2 positive cells per high power field (HPF); ++, moderate lesions/2–10 positve cells per HPF; +++, marked lesions/> 10 positive cells per HPF.

For Herts/33 strain, the most obvious difference in histological changes was in spleen in both geese and chickens. The chickens in the Herts/33-infected group presented lymphocyte depletion in the periarterial lymphatic sheath without necrosis of the spleen. No remarkable lesions were observed in the spleen of Herts/33-infected geese (Figure 5). In geese and chickens infected with Herts/33, the lesions observed in intestines were similar to those with VIId strains, but less severe at the same dpi. The histological changes of other tissues induced by Herts/33 were similar to the four VIId strains.

### Immunohistochemistry (IHC)

IHC results are presented in Table 5. The tissues of the non-infected controls were all consistently negative for NDV by IHC assay. All the five velogenic NDV strains had broad viral distribution in both geese and chickens. With all five strains, in both species, 12/13 organs (without the cerebrum) were positive.

**Figure 6:**
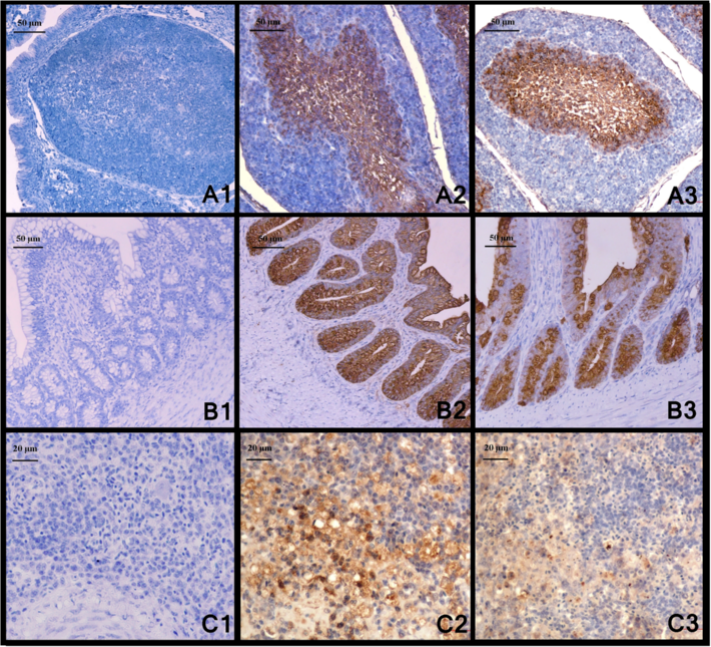
**ND viral antigen detected by IHC from tissues of geese and chickens. 6A1**: Bursa of goose from control group, and dilution buffer was used for primary incubation for negative control, 4dpi; **6A2**: IHC stain of the bursa from a goose infected with JS-5-05-Go strain showing intense positive reaction in medulla of the bursal follicles and marked degeneration of lymphocytes in the medullary region, 4dpi; **6A3**: IHC stain of the bursa from a chicken infected with JS-5-05-Go strain showing intense positive reaction in medulla of the bursal follicles and marked degeneration of lymphocytes in the medullary region, 4dpi; **6B1**: Cecum of goose from control group, and dilution buffer was used for primary incubation for negative control, 2dpi; **6B2**: IHC stain of the cecum from a goose infected with JS-5-05-Go strain presenting intense positive reaction in epithelium and glands, 2dpi; **6B3**: IHC stain of the cecum from a chicken infected with JS-5-05-Go strain presenting intense positive reaction in epithelium and glands, 2dpi; **6C1**: Spleen of goose from control group, and dilution buffer was used for primary incubation for negative control, 4dpi; **6C2**: Positive reaction in lymphocytes and macrophages in spleen of a goose infected with JS-5-05-Go, 4dpi; **6C3**: Positive reaction in lymphocytes and macrophages in spleen of a chicken infected with JS-5-05-Go, 4dpi.

All the four NDV strains of genotype VIId shared similar staining pattern and infected cell types in both chickens and geese. For lymphoid tissues, strong positive staining was mainly presented in lymphocytes and macrophages in the medulla region of the bursa follicles (Figure 6) and medulla regions of thymus, as well as lymphocytes and macrophages which were diffusely and generously scattered in spleens (Figure 6). In lymphocytes and macrophages infiltrating in mucosa and submucosa of intestine, bronchial-associated lymphoid tissue in lungs, reticulocytes in spleen and bursa, hepatocytes and Kupffer cells of liver, ND viral antigen was also detected. Moreover, positive antigen staining was also located in various types of the epithelium, including epithelium of the proventriculus and intestine, duct epithelial cell in pancreas, tubular epithelium in kidney and epithelium of the trachea. It is interesting that all the four VIId strains induced strong staining both in the crypts, gland and epithelium of intestine in geese and chickens as earlier as 2dpi (Figure 6). In general, the organs with the strongest signal were the lymphoid organs and digestive tract (Table 5), and positive antigen staining was mainly located in lymphocytes, macrophages and various types of the epithelium in these organs.

Herts/33-infected chickens had a pattern of NDV antigen distribution similar to genotype VIId strain-infected chickens. Herts/33-infected geese, on the other hand, had a lower immunohistochemical staining intensity in several organs, which often appeared at later time points when compared to genotype VIId strain-infected geese (Table 5).

## Discussion

ND is regarded as one of the most important avian diseases, causing serious economic losses in poultry worldwide. Currently, the genotype VIId NDV has been prevalent both in chicken and goose flocks in China [6,18,29]. As waterfowls are usually considered resistant to virulent NDVs, high-mortality ND outbreaks in goose flocks have drawn particular attention to the pathogenicity and origin of genotype VIId virus. Xu *et al.* postulated that goose-originated strain NA-1 of genotype VIId might have evolved from the chicken-originated strain Herts/33 of genotype IV. This hypothesis was based on phylogenic comparison of the complete sequence of the NA-1 strain with genotype I to IV strains from fowls [30]. The recent genotypes, V to VII, however, were not included in their study. The complete genome of Herts/33 was 15186 nts in the length, while the genomes of recent genotypes were 15192 nts, which excluded the evolution link between Herts/33 and genotype VII NDVs. According to the results of phylogenetic and sequence homology analysis in this paper, NA-1 strain showed higher homology with synchronic prevalent NDV isolates. On the other hand, genotype VII NDVs have been isolated frequently from chickens since the 1980s [31-33]. Therefore, it can be concluded that NA-1 strain might have been derived from an earlier VII NDV. Here, we found that the goose- and chicken-originated VIId NDVs shared very high sequence homology (Table 1) and they were intermingled and randomly distributed in the VIId cluster in the phylogenic trees (Figures 1, 2, 3). The homology among some chicken- and goose-originated NDVs even reached 100% in the HN gene sequence (Figure 1). Moreover, all the VIId NDVs from both geese and chickens shared the same pattern of characteristic amino acid substitutions different from the other genotypes, and no typical amino acid residue was detected for either goose- or chicken-originated NDVs. Therefore, it is unlikely to differentiate the chicken- and goose-originated NDVs based on their genomic sequences. We noted that Kong *et al.* developed a PCR method to distinguish between chicken- and goose-originated VIId NDVs on the basis of genomic differences they found between the two NDV groups [27]. One explanation for the discrepancy is that they used only a few goose-originated strains in their study. Two of the three goose-originated NDVs they chose to verify the validity of the test shared almost 100% sequence identity which could be considered as the same strain. Therefore, their method was not sufficiently verifiable and the results could not be reproduced well when more goose-originated NDVs were used.

Our experimental infections revealed that VIId NDVs from geese and chickens induced very similar clinical signs, pathological changes and tissue tropism in both geese and chickens,indicating that their pathobiology was correlated well with the high genomic similarity between them. However, the typical lesions induced by VIId NDVs, such as extensive necrotic foci in the spleen and multi-necrosis in intestine mucosa, were distinct from the birds infected with genotype IV virus Herts/33 isolated from chickens. Therefore, the pathogenicity and bio-characteristics of the NDVs were more closely related to their genotypes than to their origins.

From the available literatures, some genotype NDV strains showed strong host-preference. In recent years, most of the NDVs isolated from pigeons in China belong to subgenotype VIb, although the predominant subgenotype is VIId, indicating its species-preference of the VIb [34]. Similarly, the waterfowl are usually regarded as the natural host of avirulent NDVs belonging to the class I and genotype I of class II [6,34]. However, the genotype VIId NDVs isolated from the geese exhibited very similar genetic and pathobiological characteristics with the VIId viruses from the chickens in this study. Therefore, VIId NDVs either from the chicken or the geese can infect both chickens and geese equally and shows no species-preference of their original host.

Traditionally, chickens and domestic waterfowl are raised together in vast areas of China. This farming pattern may facilitate the transmission of NDVs among these domestic birds, while the VIId NDVs showed high morbidity and mortality rates both in geese and chickens. With the increasing number of waterfowl in China, ND outbreaks in waterfowl have posed a substantial threat to the poultry industry. To reduce the economic losses to the poultry industry caused by NDV, the importance of biosecurity must be emphasized, and chicken flocks should be separated far from domestic waterfowl to block the trans-species transmission between the chickens and waterfowl.

## Conclusions

Genotype VIId NDVs either from geese or chickens can infect both species and cause disease equally and no host-associated genetic and phenotypic characteristics are present between them. Therefore, active measures should be taken to prevent the trans-species transmission of VIId NDVs between the geese and chickens for the control and eradication of ND.

## Materials and methods

### Viruses

Chicken-originated NDV strain Herts/33 (genotype IV) is a kind gift from Dr. D. J. Alexander, Poultry Department, Veterinary Laboratories Agency Weybridge, England. Two chicken-originated NDV strains XJ-2/97 and JS-3-05-Ch, and two goose-originated NDV strains ZJ1 and JS-5-05-Go were isolated between 1997 and 2005 by Key Animal Infectious Disease Laboratory and identified as velogenic genotype VIId viruses [6,35]. The detailed information of these NDVs was summarized in Table 6. All of these viruses were plaque-purified for three rounds on CEF monolayers and subsequently propagated in 10-day-old SPF chicken embryos. Infective allantoic fluids containing virus were harvested and stored at −70°C until use.

**Table 6:** Background information of the NDV strains used for full-length genomic sequence analysis

**NDV strain**	**Host**	**Year of isolation**	**Accession numbers**	**Genetic grouping**	**References**
C/Ulster/67	fowl	1967	AY562991	I	
La Sota	fowl	1946	AF077761	II	[39]
Mukteswar	fowl	1941	EF201805	III	
Herts/33	fowl	1933	AY741404	IV	[40]
US/211472/02	gamefowl	2002	AY562987	V	
IF227/82	pigeon	1982	AJ880277	VI	[8]
ZJ1	goose	2000	AF431744	VIId	[7]
JS-5-05-Go	goose	2006	JN631747	VIId	
NA-1	goose	1999	DQ659677	VIId	[30]
Go/CH/HLJ/LL01/08	goose	2008	GU143550	VIId	
SF02	goose	2002	NC005036	VIId	[41]
XJ-2/97 ^a^	fowl	1997	JN618348	VIId	
JS-3-05-Ch ^a^	fowl	2005	JN618349	VIId	
GM	fowl	-	DQ486859	VIId	
FMW	fowl	2006	GU564399	VIId	
chicken/China/Guangxi11/2003	fowl	2003	DQ485231	VIId	
QH-1/79	fowl	1979	FJ751918	VIII	[7]
F48E8	fowl	1946	AY260113	IX	[38]

^a^ The genotype VIId viruses sequenced in this study.

### Eggs and birds

The source of embryonated chicken eggs was the Merial-Vital Laboratory Animal Technology Co., Ltd. specific pathogen free (SPF) White Leghorn flock. For the clinicopathologic assessment, 30-day-old SPF chickens obtained from Shandong Poultry Resarch Institute and 30-day-old geese from a local private hatchery were used. All these chickens and geese were negative for NDV antibody in hemagglutination-inhibition (HI) test [36], and kept in separated negative-pressure isolators (Model LX-III; Nanjing Labequip, Nanjing, China) of the animal facility at Yangzhou University.

### Cells

CEF monolayers were prepared with 10-day-old SPF chicken embryos as described earlier [37]. The CEFs were cultivated in medium 199 (Sigma, USA) supplemented with 4% fetal calf serum (FCS) and 1% gentamycin.

### RT-PCR amplification of viral genes and genomic sequencing

The viral genomic RNA was extracted and purified as previously described [6]. Based on the published genomic sequence of NDV strain ZJ1 [GenBank: AF431744], ten pairs of primers (data not shown) were designed to amplify the full genomic sequences of XJ-2/97 and JS-3-05-Ch strains. The 3′-and 5′- terminal ends of viral RNA were amplified as described by Qiu *et al.*[38]. PCR products were purified with the TaKaRa Agarose Gel DNA Purification Kit Ver. 2.0 (TaKaRa, Dalian, China) and sequenced by the Nanjing GenScript Biotech Co., Ltd.

### Phylogenetic and sequence analysis

On the basis of the data from NCBI, almost all the complete coding sequences of individual genes from genotype VIId NDVs of goose- and chicken-origin without recombination were collected and analyzed. In this study, 150 complete HN gene sequences (40 from goose isolates and 110 from chicken isolates), 98 complete F gene sequences (18 from goose isolates and 80 from chicken isolates), 28 complete P gene sequences (6 from goose isolates and 22 from chicken isolates), 21 complete NP gene sequences (6 from goose isolates and 15 from chicken isolates), 19 complete M gene sequences (5 from goose isolates and 14 from chicken isolates), and 13 complete L gene sequences (5 from goose isolates and 8 from chicken isolates) were used for analysis (Additional file 2, Additional file 3, Additional file 4, Additional file 5, Additional file 6 and Additional file 7). The prediction of amino acid sequences, alignment of sequences, and phylogenetic analysis were performed using MegAlign in the Lasergene package (DNASTAR Inc. Madison, WI, U.S.). Phylogenetic trees were created using the neighbor-joining method and MEGA 5.0 based on nucleotide sequences of the coding regions of F and HN genes, respectively. The complete genomic sequences of 18 NDV strains (Table 6) from genotypes I through IX were also included for analysis [7,8,30,38-41].

### Infection of CEF with different NDVs

CEF cells were infected with chicken NDV strain Herts/33 of genotype IV, chicken-originated NDV strain XJ-2/97, JS-03-05-Ch, and goose-originated NDV strain ZJ1, JS-5-05-Go of genotype VIId respectively. For all viruses, a multiplicity of infection (MOI) of 0.01 based on infectious virus particle concentration determined as tissue-culture infectious dose (TCID_50_/mL) was used. Comparative investigations and HA test of supernatant fluid were carried out at 12-hour intervals after inoculation. The HA test was performed with 1% chicken erythrocytes as described by the OIE [10]. The formation of syncytium was assessed at 48 hpi by blinded observers using Giemsa staining as described by Ritter *et al.*[42].

### Clinicopathologic assessment

Seventy-two 30-day-old SPF chickens were randomly divided into six groups, 12 chickens each. Five groups were infected with four genotype VIId NDV strains: XJ-2/97, JS-03-05-Ch, ZJ1, and JS-5-05-Go, and the genotype IV NDV strain Herts/33, respectively. Each chicken was inoculated with a total dose of 10^5.0^ 50% embryo infectious doses (EID_50_) in 0.1 ml of virus *via* the nasal, oral and ocular routes, except for the sixth group, which was inoculated with phosphate buffered saline (PBS) by the same route as a negative control. In the goose experiment, seventy-two 30-day-old geese were divided into six groups and infected with different NDV strains as described in chickens mentioned above.

After inoculation, the geese and chickens were clinically monitored every day for a week. Two geese and two chickens of each group were euthanatized with an intravenous injection of pentobarbital at 2, 4 and 7 dpi. The seriously diseased birds were euthanatized regardless of the scheduled sampling day. The experiment with chickens was terminated at 5 dpi, because of sever morbidity and mortality in NDV-infected groups. The experiment with geese was terminated at 7 dpi. The survivors and control geese were euthanatized at 7 dpi. Immediately post-mortem, necropsies were performed on the geese and chickens using a standard protocol [43]. All animal work was approved by the Jiangsu Administrative Committee for Laboratory Animals (Permission number: SYXK-SU-2007-0005). Fresh tissue samples of the spleen, trachea, proventriculus, liver, lung, pancreas, small intestine (duodenum and ileum), large intestine (cecum), thymus, bursa of Fabricius, kidney, and cerebrum were collected from chickens and geese sequentially. Collected tissues were fixed by immersion in 10% neutral buffered formalin. All samples were embedded into paraffin, and sectioned at 3 μm and then routinely deparaffinized and stained with hematoxylin and eosin for histopathological examination [44].

For IHC analysis, deparaffinized and hydrated sections were soaked in 0.3% H_2_O_2_(v/v) in 100% methanol for 30 minutes at room temperature, The sections were subjected to antigen retrieval by microwaving for 10 minutes at full power in 0.1 M citrate buffer, followed by blocking with 1% bovine serum albumin (BSA) in PBS (pH 7.4). Then slides were incubated in primary antibody (a mouse-derived monoclonal antibody against NDV F protein which produced by Hu *et al.*[45]) diluted 1:400 in PBST (PBS with 0.05% Tween20) containing 0.1% BSA for 2 hours at 37°C. After washing, sections were incubated with secondary antibody (peroxidase conjugated goat-anti-mouse IgG (Sigma, St Louis, MO, U.S.)) diluted 1:1000 at 37°C for 1 hour. Substrate development was with 3,3′-diaminobenzidine (DAB) (Boster, Wuhan, China). Sections were counterstained lightly with hematoxylin. For each tissue specimen in our study, a duplicate set of tissue sections were also prepared using the same steps, but omitting the incubation with the primary antibody for control. A semi-quantitative scoring of IHC staining was conducted as previously described [44].

## Competing interests

The authors declare that they have no competing interests.

## Authors’ contributions

YW carried out the study design, phylogenetic analysis, sequence alignment, pathological comparison, and drafted the manuscript. ZD contributed for RNA preparation and RT-PCR. YK, LZ participated in the whole procedure of the animal experiment. XBW contributed for histological examination. SH, QQS, QS, XW and YW contributed to the design of the study and revision of the manuscript. XL conceived of the study, provided consultation and coordination, and helped to draft the manuscript. All authors read and approved the final manuscript.

## Supplementary Material

Additional file 1 **Gross lesions on other organs of chickens and geese infected with genotype VIId NDV strains.** 7A: Severe hemorrhage and necrosis in the intestinal tract of goose infected with JS-5-05-Go, 4 dpi; B1: Hemorrhage on the thymus of goose infected with JS-5-05-Go, 4dpi; B2: Sever lymphoid necrosis of thymus in the histology from a goose infected with JS-5-05-Go, 4dpi. C: Obvious necrosis in the pancreas of goose infected with JS-5-05-Go, 4dpi. D: Pale kidneys with deposition of urate from a chicken infected with JS-5-05-Go, 4dpi. E: Hemorrhage and edema of proventriculus of chicken infected with JS-5-05-Go, 4dpi. Click here for file

Additional file 2 Background information of the 21 NP sequences investigated in the study. Click here for file

Additional file 3 Background information of the 28 P sequences investigated in the study. Click here for file

Additional file 4 Background information of the 19 M sequences investigated in the study. Click here for file

Additional file 5 Background information of the 98 F sequences investigated in the study. Click here for file

Additional file 6 Background information of the 150 HN sequences investigated in the study. Click here for file

Additional file 7 Background information of the 13 HN sequences investigated in the study. Click here for file
