# Supplementary figures and images for: Lack of detection of host associated differences in Newcastle disease viruses of genotype VIId isolated from chickens and geese
Source: Virol J. 2012 Sep 13;9:197. doi: 10.1186/1743-422X-9-197 (PMC3491030; doi:10.1186/1743-422X-9-197)

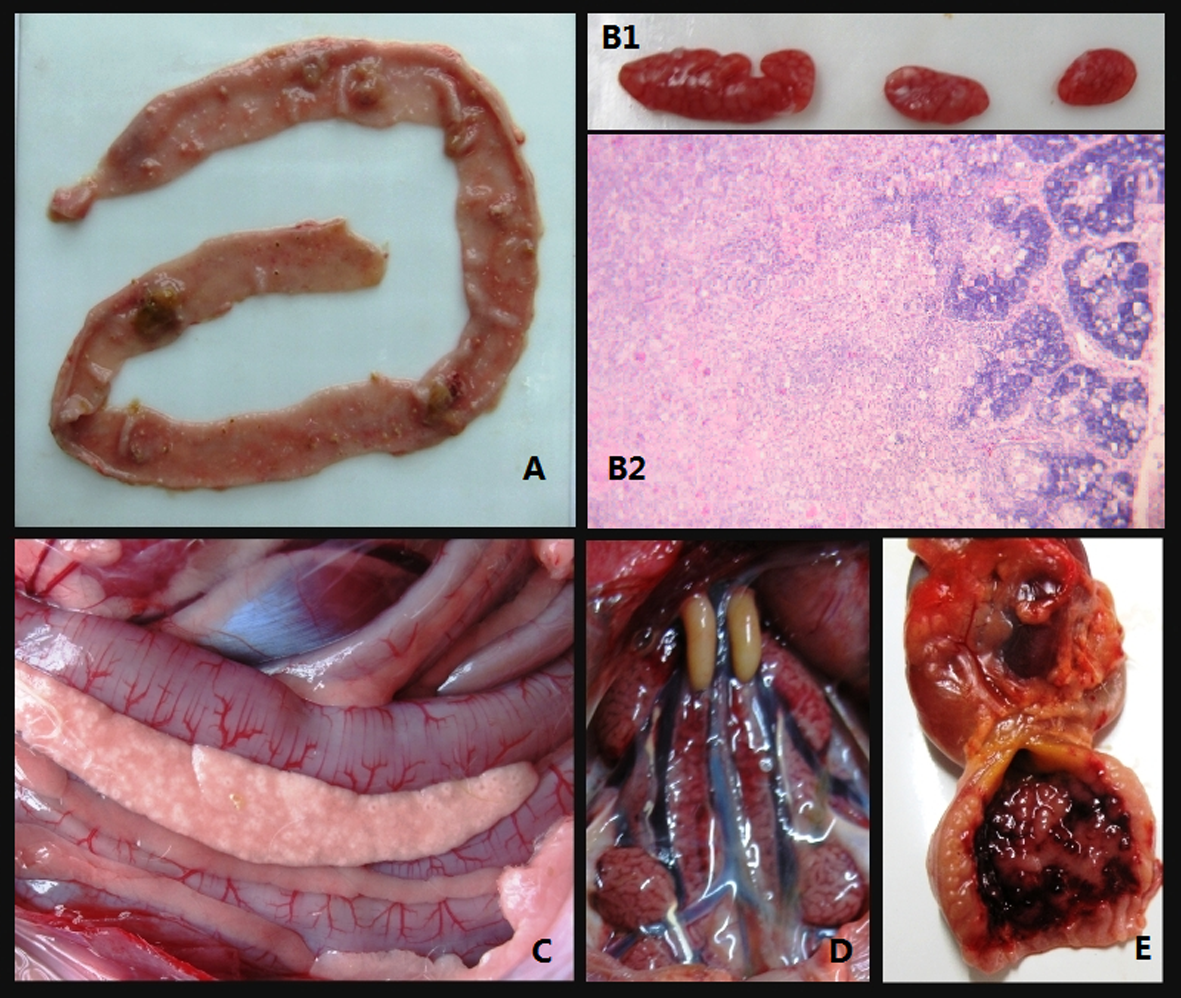

Supplement: Additional file 1 — Gross lesions on other organs of chickens and geese infected with genotype VIId NDV strains. 7A: Severe hemorrhage and necrosis in the intestinal tract of goose infected with JS-5-05-Go, 4 dpi; B1: Hemorrhage on the thymus of goose infected with JS-5-05-Go, 4dpi; B2: Sever lymphoid necrosis of thymus in the histology from a goose infected with JS-5-05-Go, 4dpi. C: Obvious necrosis in the pancreas of goose infected with JS-5-05-Go, 4dpi. D: Pale kidneys with deposition of urate from a chicken infected with JS-5-05-Go, 4dpi. E: Hemorrhage and edema of proventriculus of chicken infected with JS-5-05-Go, 4dpi. [file 1743-422X-9-197-S1.tiff]
